# Shrinkage, Degree of Conversion, Water Sorption and Solubility, and Mechanical Properties of Novel One-Shade Universal Composite

**DOI:** 10.3390/polym17202728

**Published:** 2025-10-11

**Authors:** Long Ling, Theresa Lai, Pei-Ting Chung, Raj Malyala

**Affiliations:** Glidewell Dental, Irvine, CA 92616, USA

**Keywords:** one-/single-shade universal composites, shrinkage, degree of conversion, water sorption and solubility, flexural strength, diametral tensile strength

## Abstract

This study aims to evaluate the shrinkage, degree of conversion, water sorption and solubility, and mechanical properties of a newly developed one-shade universal composite and compare it with five other commercially available universal composites with one or multiple shades. Our proprietary resin and filler technologies developed the experimental one-shade universal composite (Experimental). Volumetric shrinkage was determined using the AcuVol video imaging method (n = 5). Degree of conversion was measured using FTIR (n = 5). Water sorption and solubility (15 × 1 mm, n = 5) and flexural strength and modulus (2 × 2 × 25 mm, n = 5) were measured according to ISO-4049. Diametral tensile strength (6 × 3 mm, n = 8) was tested according to ANSI/ADA-Specification #27. The data were analyzed using one-way ANOVA and post hoc Tukey tests (*p* ≤ 0.05). Like Clearfil Majesty ES-2, Experimental showed lower or significantly lower volumetric shrinkage than other composites. Experimental exhibited a considerably higher degree of conversion and high flexural modulus compared to the others. However, there are no significant differences in flexural strength among these universal composites except for Omnichroma. Experimental also displayed significantly higher diametral tensile strength than the others, except similar to Filtek Supreme Ultra. Experimental has the lowest values of water sorption and solubility among the composites tested. The experimental universal composite demonstrated improved or comparable physical and mechanical properties compared to commercially available one-shade universal composites or multi-shade conventional universal composites, which is of significance for the clinical performance of dental restorations.

## 1. Introduction

Resin-based composites are widely used in dentistry as direct restorative materials for various applications due to their improved esthetics, ease of use, good bonding to tooth structure, improved mechanical properties, and advancements in composite technology [1,2,3,4,5]. Universal composite is the most popular category of resin composites, which can be used for both posterior and anterior restoration or all classes (I–VI) of dental caries or cavities. For an excellent esthetic outcome, manufacturers provided multiple (as many as 16, according to Vita Classical) enamel/dentin/body shades for most composites in the market, which complicates shade selection and matching for the dentists’ daily practice. Additionally, dentists need to keep various shades in stock. To simplify the restorative workflow and reduce inventory and expired composites for both dentists and manufacturers, some dental manufacturers have developed their composites with reduced shades (from 16 Vita shades) to match all clinical situations, for example, Filtek Universal Restorative (3M, 8 shades) and TPH Spectra ST (Dentsply, 5 shades). Notably, Tokujama first introduced Omnichroma, a one-shade (or single-shade) universal composite, to cover all Vita classical shades in recent years, utilizing “The Smart Chromatic Technology” [6]. This technology uses uniformly sized 260 nm spherical fillers of zirconia-silica, which generate a red-to-yellow structural color. Later, other dental companies also launched similar one-shade or three-shade universal composites using different technologies, such as Kuraray (Clearfil Majesty ES-2, Light Diffusion Technology), Kulzer (Venus Diamond/Venus Pearl, Adaptive Shade Matching Technology), and Kerr (SimpliShade, Adaptive Response Technology). The most recent studies have focused on the one-shade universal composites’ color, optical, and esthetic behavior due to their great advantages in various clinical situations [7,8,9,10,11,12,13,14,15,16]. As one-shade universal composites are developed to simplify the shade selection process while maintaining esthetic outcomes, there is a concern about physical and mechanical properties like polymerization shrinkage, degree of conversion, water sorption and solubility, and mechanical properties, which are crucial for composite restorations to the tooth structure and affect their clinical performance, especially for clinical durability and longevity. However, only a few studies have been conducted to evaluate the physical and mechanical performance of these newly introduced one-shade universal composites in this area.

The objective of this study is to develop a new one-shade universal composite with favorable physical and mechanical properties and compare it with other commercially available universal composites with one, fewer, or multiple shades. The hypothesis is that this new one-shade universal composite has improved or comparable physical and mechanical properties compared to commercially available one-shade universal composites or multi-shade conventional universal composites.

## 2. Materials and Methods

### 2.1. Materials

A new one-shade universal composite (Experimental or Exp) was developed by our proprietary resin and filler technology, which contains resins (like bisphenol A glycidyl methacrylate and triethyleneglycol dimethacrylate), initiator (like camphorquinone), additives (like 2,6-di-(*tert*-butyl)-4-methylphenol), pigments (like red and yellow), and fillers (like barium glass). All resin monomers and additives were mixed with an overhead stirrer (IKA RW20 digital, Wilmington, NC, USA) to form a homogeneous resin mixture. The resulting resin mixtures were further mixed with fillers using Speedmixer (Hauschild, DAC 150.1, Hamm, Germany) until a uniform composite paste was formed. Five other commercially available universal composites with one or multiple shades, Omnichroma (Tokuyama, Tokyo, Japan), Clearfil Majesty ES-2 (Kuraray, Tokyo, Japan), SimpliShade (Kerr, Orange, CA, USA), Filtek Supreme Ultra (3M, St. Paul, MN, USA), and TPH Spectra ST (Dentsply, York, PA, USA), were selected for comparison in this study. Further information about the universal composites studied is shown in Table 1.

### 2.2. Volumetric Shrinkage

An AcuVol 2 instrument (Bisco, Inc., Schaumburg, IL, USA) was used to test volumetric shrinkage (%) according to the literature [17]. Each composite (about 25 mg) was carefully placed onto the sample holder’s base to form a bubble-free, ball specimen and then light-cured for 20 s at a light intensity of 1100 mW/cm^2^ (Bluephase Style, Ivoclar Vivadent AG, Schaan, Liechtenstein). As five minutes was enough for the volumetric shrinkage to stabilize after light curing, the volumetric shrinkage of each composite material (n = 5) was obtained at five minutes after light curing for twenty seconds.

### 2.3. Degree of Conversion (DC)

A Bruker ALPHA FTIR spectrometer with a Diamond Crystal ATR (Bruker, Billerica, MA, USA) was used to measure the degree of conversion based on the literature [18]. Disk specimens (n = 5) filled in the mold (10 mm in diameter × 1 mm in thickness) were placed directly on the diamond crystal plate and light-cured for 40 s in situ with a light intensity of approximately 1100 mW/cm^2^ and a broadband spectrum of 385–515 nm (Bluephase Style, Ivoclar Vivadent AG, Schaan, Liechtenstein). The spectra of the uncured and cured composites were recorded at a wavelength range of 4000–400 cm^−1^, a resolution of 4 cm^−1^, and a sample scan time of 24 s.

The DC was determined based on the peak heights of aliphatic double-bond absorption around 1638 cm^−1^ and of the aromatic double bond around 1607 cm^−1^ (as the internal standard) as follows:

DC (%) = [1 − (A_p_/A_p0_)/(A_m_/A_m0_)] × 100

where A_p_ is the peak height of the cured composite (polymer), A_m_ is the peak height of uncured composite (monomer) at 1638 cm^−1^, and A_m0_ and A_p0_ are the peak heights at 1607 cm^−1^ before and after curing, respectively.

### 2.4. Water Sorption and Solubility

The water sorption and solubility tests were measured according to ISO 4049 (2009). The disk specimens [(15.0 ± 0.1) mm in diameter and (1.0 ± 0.1) mm in thickness, n = 5] were prepared in a Teflon mold and light-cured with a light intensity of approximately 1100 mW/cm^2^ (Bluephase Style, Ivoclar Vivadent AG, Schaan, Liechtenstein) for 20 s at five locations to cover the whole disk. The cured specimens were stored in a desiccator at 37 °C for 22 h, transferred to another desiccator at 23 °C for 2 h, and weighed until a constant mass (m_1_) was obtained by repeating this cycle. The specimens were then stored in deionized water at 37 °C for 7 days. The surface water on the specimen was blotted away to free it from visible moisture and then waved in the air for 15 s. Then, the mass m_2_ of the specimens was recorded. The specimens were reconditioned in a desiccator and weighed until a constant weight (m_3_) was obtained using the cycle described above.

Water sorption: W_sp_ = (m_2_ − m_3_)/V

Solubility: W_sl_ = (m_1_ − m_3_)/V

where V is the volume of the specimen.

### 2.5. Flexural Strength and Flexural Modulus

The flexural strength and modulus were tested using the three-point bending method in accordance with the literature [19] based on ISO-4049 (2009). Bar-shaped specimens (thickness × width × length = 2 × 2 × 25 mm, n = 5) were prepared from composite materials [light-cured for 40 s at three spots on both sides with a Bluephase Style curing gun (see above)] and tested under a crosshead speed of 0.75 mm/min on an Instron 5564 universal testing machine after storage in de-ionized water at 37 °C for 24 h. The flexural modulus was determined from the slope of the linear region of the stress–strain curve.

### 2.6. Diametral Tensile Strength

The disk specimens (diameter × height = 6 × 3 mm, n = 8) were made via light curing for 40 s on the top and bottom using the Bluephase Style curing gun (same as above). Their diametral tensile strength was determined according to ANSI/ADA- Specification #27 [20] under a testing speed of 1.0 mm/min on a Shimadzu (AGS-X-10 KN-table top, Kyoto, Japan) universal testing machine.

### 2.7. Statistical Analysis

One-way ANOVA was performed to determine the differences among the 6 groups/materials of each property (volumetric shrinkage, degree of conversion, water sorption and solubility, flexural strength, flexural modulus, and diametral tensile strength), followed by Tukey’s post hoc comparison with a confidence level of 0.05. The statistical analysis was performed with Minitab 21 Statistical Software (Minitab, LLC., State College, PA, USA).

## 3. Results

**Figure 1:**
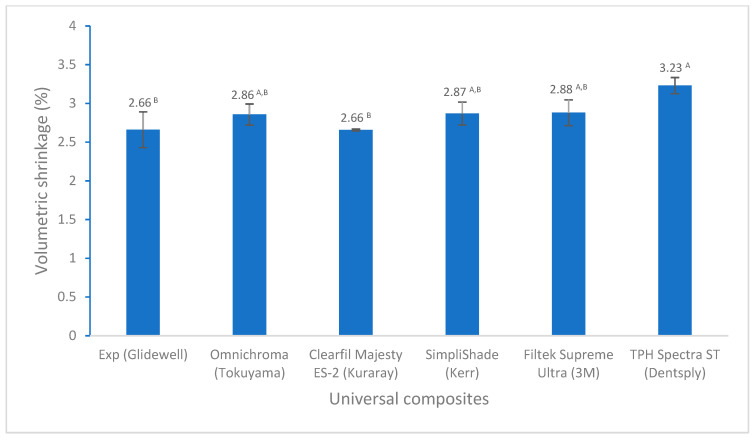
Volumetric shrinkage of universal composites (values with the same superscript are not significantly different (*p* > 0.05)).

**Figure 2:**
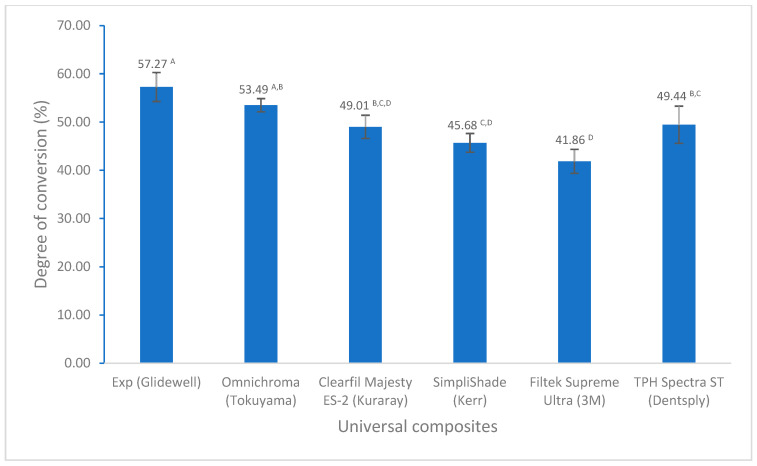
Degree of conversion of universal composites (values with the same superscript are not significantly different (*p* > 0.05)).

**Figure 3:**
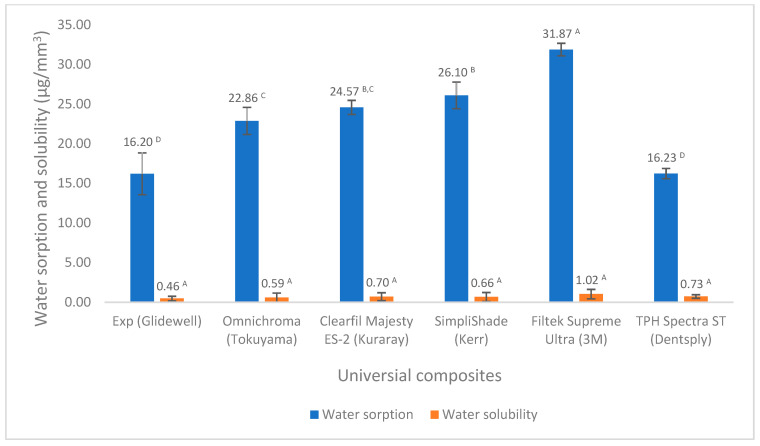
Water sorption and solubility of universal composites (values with the same superscript are not significantly different (*p* > 0.05)).

There are not many differences in volumetric shrinkage among these universal composites, except for TPH Spectra ST. However, both Experimental and Clearfil Majesty ES-2 have the lowest value (Figure 1). The experimental universal composite exhibited a significantly higher degree of conversion compared to the others (Figure 2). Although all composites meet the ISO standard of the water sorption and solubility (water sorption ≤ 40 µg/mm^3^, solubility ≤ 7.5 µg/mm^3^), Experimental has the lowest values of water sorption and solubility among the composites tested in this study (Figure 3).

**Figure 4:**
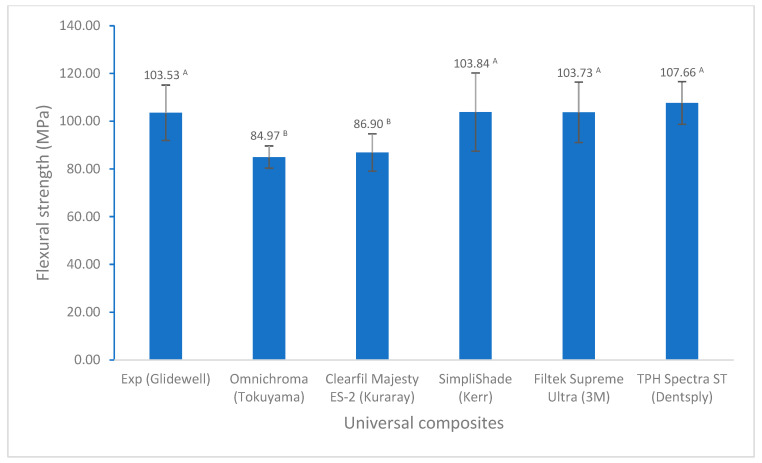
Flexural strength of universal composites (values with the same superscript are not significantly different (*p* > 0.05)).

**Figure 5:**
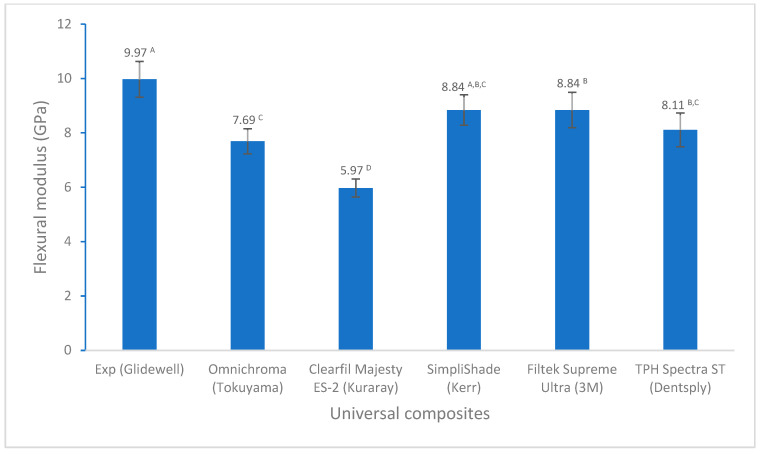
Flexural modulus of universal composites (values with the same superscript are not significantly different (*p* > 0.05)).

**Figure 6:**
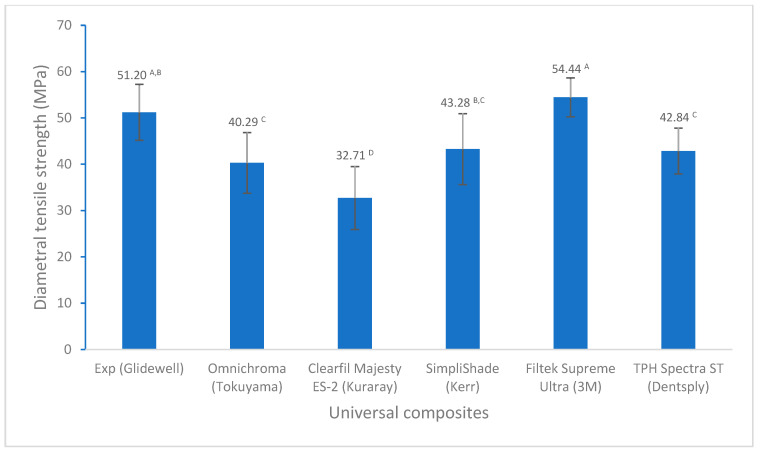
Diametral tensile strength of universal composites (values with the same superscript are not significantly different (*p* > 0.05)).

For flexural properties, there are no significant differences in flexural strength among these universal composites except for Omnichroma (Figure 4). However, Experimental showed a significantly higher flexural modulus than other universal composites (Figure 5). In terms of tensile strength, Experimental displayed significantly higher diametral tensile strength than the others, except similar to Filtek Supreme Ultra (Figure 6).

## 4. Discussion

Polymerization shrinkage is still a major reason for clinical failure of dental composite restorations, as the shrinkage and associated stress cause debonding and marginal leakage between the composite restorations and the tooth structure, post-operative sensitivity, etc., eventually leading to secondary caries [21,22,23,24]. Therefore, a reduced shrinkage is highly desirable for dental composite restorations. There have been various methods to measure the volumetric shrinkage of resin-based composites, such as mercury dilatometry, the Archimedes method, and the video imaging method [18,22,25,26,27,28,29]. The AcuVol video imaging method was used to measure the volumetric shrinkage of universal composites in this study, as AcuVol is a simple noncontact imaging analysis tool for volume changes in small sample amounts in real time [18,28].

The volumetric shrinkage of all universal composites tested varies between 2.66 and 3.23 vol% (Figure 1), which is in good agreement with the results of same method (AcuVol) or other methods like mercury dilatometry for commercial composites between 2 and 4 vol% [30,31,32]. This is because most dental resin composites consist of dimethacrylate monomers as structural monomers and diluent monomers, such as bisphenol A diglycidyl ether dimethacrylate (BisGMA), urethane dimethacrylate (UDMA), and triethyleneglycol dimethacrylate (TEGDMA). All universal composites tested exhibited similar volumetric shrinkage, except for TPH Spectra ST, which had the highest value. The reason is probably that some resins and fillers used in these composites are the same or similar; for example, most composites contain ethoxylated bisphenol A dimethacrylate (BisEMA) (Exp, SimpliShade, Filtek Supreme Ultra, and TPH Spectra ST) and micro-barium silica (Exp, Clearfil Majesty ES-2, SimpliShade, and TPH Spectra ST) (Table 1), and the filler loading is also similar at 78–79 wt.%. The difference in composite composition (resin monomers and fillers) made these composites show slightly different volumetric shrinkage. Unfortunately, the manufacturers only provided limited information on the composition of resins and fillers, which are the major factors affecting the volumetric shrinkage of resin composites [18,31]. The experimental composite utilized low-shrinkage monomers, such as BisGMA, BisEMA, nano-silica, and nano-ytterbium fluoride to reduce volumetric shrinkage, resulting in the lowest value of volumetric shrinkage (2.66%). Clearfil Majesty ES-2 also has the lowest volumetric shrinkage (2.66%). The pre-polymerized organic filler used in Clearfil Majesty ES-2 probably made some contributions to its low volumetric shrinkage, as pre-polymerized filler can reduce polymerization shrinkage [30].

The degree of conversion significantly affects the properties of resin composites, like physical, mechanical, and biocompatible properties [18,33,34,35,36]. Higher DC generally leads to improved mechanical properties, while lower DC can result in increased monomer elution, potential biocompatibility issues, and reduced restoration longevity. The degree of conversion can be measured with a couple of testing methods [18]. FTIR is the most extensively utilized approach for measuring the DC in most research due to its sensitivity to specific functional groups, such as aliphatic double bonds involved in polymerization reactions [18]. The mean values of degree of conversion for all universal composites tested are from 42% to 57% (Figure 2), which fall within the typical range of methacrylate-based resin composites from 40 to 75% depending on the composite composition, polymerization conditions (light source, irradiance, etc.), post-curing, and used testing methods [18,34,37,38]. For example, the DC of Omnichroma (53.49%) in this study is similar to the one reported by Atali et al. (52.09% by FTIR) [39]. However, the experimental universal composite exhibited a significantly higher degree of conversion than the others (Figure 2). This is primarily caused by the composition of composites, particularly the chemical structure and composition of the resins, including monomers, initiators, inhibitors, etc. [34,37,39,40,41,42]. Although some of the monomers in these composites are the same, such as BisEMA and TEGDMA, the monomer proportion in different composites may be different, resulting in various values in DC. In addition, the dual initiator system used in the experimental composite, i.e., highly active photoinitiator [bis(2,4,6-trimethybenzoyl)-phenylphosphine oxide] and camphorquinone, also made some contributions to the higher DC of the experimental composite [41,42,43], which is in good agreement with our previous results [42].

**Figure 7:**
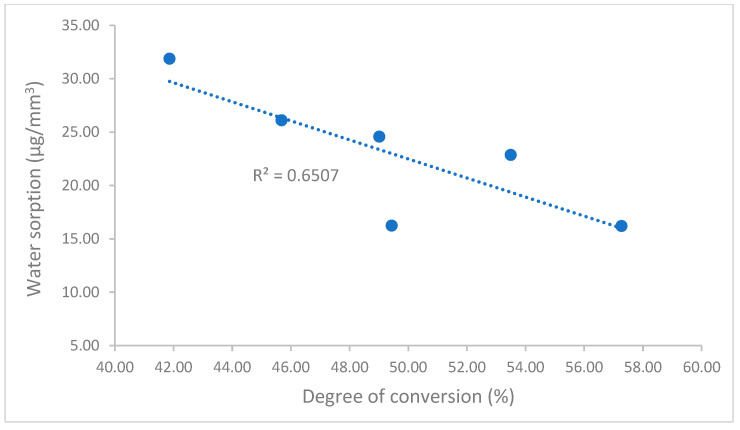
Correlation of degree of conversion and water sorption.

**Figure 8:**
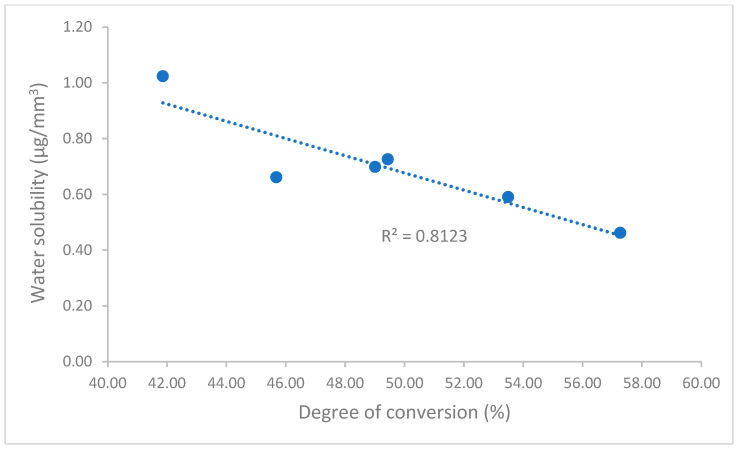
Correlation of degree of conversion and water solubility.

As dental resin composites may absorb water or dissolve in water when exposed to oral fluids, which can lead to expansion and potentially leach out unreacted monomers, water sorption and solubility significantly impact resin composites’ dimensional stability, biocompatibility, mechanical strength, and so on [44,45,46,47]. Water sorption and solubility for all composites are in the ranges of 16.20–31.87 µg/mm^3^ and 0.46–1.02 µg/mm^3^, respectively (Figure 3), with both passing the ISO 4049 standard for water sorption (≤40 µg/mm^3^) and solubility (≤7.5 µg/mm^3^). However, Experimental showed significantly lower water sorption than the other composites, except similarly to TPH Spectra ST, and lower water solubility than all other composites. These different values of water sorption and solubility are mainly attributed to the composite composition and the degree of conversion [45,48,49,50]. Although the manufacturers did not provide detailed information on the composition of these universal composites, the degree of conversion, which is affected by the composition of the composites, can reflect and influence these differences. A higher degree of conversion can reduce unreacted monomers, resulting in a decrease in water sorption and solubility. This was demonstrated in this study through the strong correlation between the degree of conversion and water sorption (Pearson’s correlation coefficient, R = 0.81) and solubility (R = 0.90) (Figure 7 and Figure 8).

The flexural strength and flexural modulus are important mechanical properties and are widely used to evaluate the mechanical behavior of dental resin composites, as they affect the dental composites’ ability to withstand chewing forces and other stresses. Therefore, both are crucial for the long-term success of dental restorations. Flexural strength represents the maximum stress a material can withstand before breaking under a bending load, while the flexural modulus indicates its stiffness or resistance to deformation during bending. According to the ISO standards (ISO 4049), the flexural strength of direct resin composites is required to not be less than 80 MPa. The flexural strength of all universal composites in this study is greater than 80 MPa (Figure 4). However, Omnichroma and Clearfil Majesty ES-2 showed lower values of flexural strength and flexural modulus, and the experimental composite (Exp) had a significantly higher flexural modulus than other universal composites (Figure 4 and Figure 5). This could be attributed to the composite composition, i.e., different resin monomers and fillers. For example, Omnichroma used 260 nm uniformly sized spherical fillers with greater constant interparticle spacing, and crack propagation tended to be simple and easy, leading to low fracture resistance and lower flexural strength [51], which is supported by the result reported by Atali et al. (82.79 ± 18.59 MPa based on ISO 4049) [39]. The urethane dimethacrylate (UDMA) and triethyleneglycol dimethacrylate (TEGDMA) with a low modulus (compared to Bisphenol A diglycidyl ether dimethacrylate (BisGMA) used in Omnichroma probably also made some contributions to the flexural strength and flexural modulus of Omnichroma [52]. Pre-polymerized organic filler in Clearfil Majesty ES-2 may be a consequence of the lower flexural strength and flexural modulus, as the incorporation of pre-polymerized filler particles in composites may result in a reduction in mechanical properties, especially for pre-polymerized organic fillers, which also depends on the pre-polymerized filler’s composition, loading, etc. [30,53]. The higher flexural strength and flexural modulus of the experimental composite (Exp) may be attributed to non-aggregated silica (80 nm), BisGMA, strong interactions between monomers and fillers, and a higher degree of conversion [51,52].

Diametral tensile strength measures a material’s resistance to pulling or stretching forces when subjected to a compressive force applied along the diameter of a cylindrical specimen, which creates tensile stress in the material. Diametral tensile strength can indicate how well a restorative material will withstand the tensile forces experienced in the mouth during chewing and biting. In general, diametral tensile strength has strong correlation with flexural strength and flexural modulus, respectively [19,54], which is also supported by our results in the present study (R = 0.6728 and 0.8728 for diametral tensile strength and flexural strength, and flexural modulus, respectively). Like with the flexural strength and flexural modulus, Omnichroma and Clearfil Majesty ES-2 exhibited lower diametral tensile strength. Experimental showed diametral tensile strength similar to that of Filtek Supreme Ultra and significantly higher strength than that of the other composites (Figure 6). This may also be a consequence of different composite compositions, as mentioned above.

## 5. Conclusions

As a newly developed one-shade universal composite, the experimental universal composite displayed lower volumetric shrinkage, higher degree of conversion, lower water sorption and solubility, favorable flexural strength and flexural modulus, and diametral tensile strength compared to other commercially available one-shade or multiple-shade universal composites. Therefore, the study hypothesis has been proven. The experimental universal composite is suitable for use in dental direct restorations with potentially better clinical performance.

## Figures and Tables

**Table 1:** Universal composites used.

Universal Composite	Manufacturer	Shade	Resin	Filler	Filler Content (w%)
Exp	Glidewell	One	BisGMA, BisEMA, UDMA, TEGDMA,	Nano-silica and micro-barium glass, Ytterbium fluoride	78
OmniChroma	Tokuyama	One	UDMA, TEGDMA	Spherical nano-silica-zirconia	79
Clearfil Majesty ES2	Kuraray	One (Posterior)	BisGMA, Aromatic- and Aliphatic-dimethacrylates	Barium glass, Pre-polymerized organic filler	78
SimpliShade	Kerr	Three	BisEMA, TEGDMA	Zirconium oxide, Silicon dioxide, Ytterbium Fluoride	81
Filtek Spreme Ultra	3M ESPE	Multiple	BisGMA, UDMA, BisEMA(6), TEGDMA	Nano-zirconia and nano-silica and zirconia/silica cluster	78.5
TPH Spectra ST(HV)	Dentsply	Multiple	Poly-UDMA, BisEMA, TEGDMA, Methacrylic polysiloxane	Barium glass, Pre-poly-merized filler, Ytterbium fluoride	79

BisGMA—bisphenol A diglycidyl ether dimethacrylate; UDMA—urethane dimethacrylate; TEGDMA—triethyleneglycol dimethacrylate; BisEMA—ethoxylated bis phenol A dimethacrylate.

## Data Availability

Data are contained in this article. The original contributions presented in this study are included in the article material. Further inquiries can be directed to the corresponding author.

## References

[1] Alzraikat H., Burrow M.F., Maghaireh G.A., Taha N.A. (2018). Nanofilled resin composite properties and clinical performance: A review. Oper. Dent..

[2] Yadav R., Kumar M. (2019). Dental restorative composite materials: A review. J. Oral Biosci..

[3] Liu J., Zhang H., Sun H., Liu Y., Liu W., Su B., Li S. (2021). The Development of filler morphology in dental resin composites: A review. Materials.

[4] Ilie N., Ionescu A.C., Diegelmann J. (2022). Characterization of universal chromatic resin-based composites in terms of cell toxicity and viscoelastic behavior. Dent. Mater..

[5] Ferracane J.L. (2024). A historical perspective on dental composite restorative materials. J Funct Biomater..

[6] Tokujama Technical Report-OMNIchroma. https://omnichroma.com/us/wp-content/uploads/sites/4/2019/01/OMNI-Tech-Report-Color-Final.pdf.

[7] de Abreu J.L.B., Sampaio C.S., Jalkh E.B., Hirata R. (2020). Analysis of the color matching of universal resin composites in anterior restorations. J. Esthet. Restor. Dent..

[8] Islam M.S., Huda N., Mahendran S., Ac S.A., Nassar M., Rahman M.M. (2023). The blending effect of single-shade composite with different shades of conventional resin composites—An in vitro study. Eur. J. Dent..

[9] Sanchez N.P., Powers J.M., Paravina R.D. (2019). Instrumental and visual evaluation of the color adjustment potential of resin composites. J. Esthet. Restor. Dent..

[10] Baghizadeh S., Tabari K., Abbasi K., Farnaz S., Tabatabaei S.F., Heshmat H. (2024). Assessing shade matching capability of Omnichroma, a single shade composite in posterior restorations: An in vitro study. J. Med. Life.

[11] Lucena C., Ruiz-López J., Pulgar R., Bona A.D., Pérez M.M. (2021). Optical behavior of one-shaded resin-based composites. Dent. Mater..

[12] Anwar R.S., Hussein Y.F., Riad M. (2024). Optical behavior and marginal discoloration of a single shade resin composite with a chameleon effect: A randomized controlled clinical trial. BDJ Open.

[13] Kobayashi S., Nakajima M., Furusawa K., Tichy A., Hosaka K., Tagami J. (2021). Color adjustment potential of single-shade resin composite to various-shade human teeth: Effect of structural color phenomenon. Dent. Mater. J..

[14] Khayat W.F. (2024). In vitro comparison of optical properties between single-shade and conventional composite resin restorations. Cureus.

[15] Durand L.B., Ruiz-López J., Perez B.G., Ionescu A.M., Carrillo-Pérez F., Ghinea R., Pérez M.M. (2021). Color, lightness, chroma, hue, and translucency adjustment potential of resin composites using CIEDE2000 color difference formula. J. Esthet. Restor. Dent..

[16] Batista G.R., Borges A.B., Zanatta R.F., Pucci C.R., Torres C.R.G. (2023). Esthetical properties of single-shade and multishade composites in posterior teeth. Int J Dent..

[17] Ling L., Taremi N., Malyala R. (2022). A novel low-shrinkage resin for 3D printing. J. Dent..

[18] Ling L., Chen Y., Malyala R. (2024). Assessment of degree of conversion and volumetric shrinkage of novel self-adhesive cement. Polymers.

[19] Ling L., Ma Y., Malyala R. (2021). A novel CAD/CAM resin composite block with high mechanical properties. Dent Mater..

[20] (2019). Standard Test Method for Vickers Indentation Hardness of Advanced Ceramics.

[21] Chung C.M., Kim J.G., Kim M.S., Kim K.M., Kim K.N. (2002). Development of a new photocurable composite resin with reduced curing shrinkage. Dent. Mater..

[22] Weinmann W., Thalacker C., Guggenberger R. (2005). Siloranes in dental composites. Dent. Mater..

[23] Venkatesh A., Saatwika L., Karthick A., Subbiya A. (2020). A review on polymerization shrinkage of resin composites. Eur. J. Mol. Clin. Med..

[24] Eick J.D., Welch F.H. (1986). Polymerization shrinkage of posterior composite resins and its possible influence on postoperative sensitivity. Quintessence Int..

[25] Oberholzer T.G., Grobler S.R., Pameijer C.H., Rossouw R.J. (2002). A modified dilatometer for determining volumetric polymerization shrinkage of dental materials. Meas. Sci. Technol..

[26] Nitta K., Nomoto R., Tsubota Y., Tsuchikawa M., Hayakawa T. (2017). Characteristics of low polymerization shrinkage flowable resin composites in newly developed cavity base materials for bulk filling technique. Dent. Mater. J..

[27] (2013). Dentistry-Polymerization Shrinkage: Method for Determination of Polymerization Shrinkage of Polymer-Based Restorative Materials.

[28] Sharp L.J., Choi I.B., Lee T.E., Sy A., Suh B.I. (2003). Volumetric shrinkage of composites using video imaging. J. Dent..

[29] Tiba A., Charlton D.G., Vandewalle K.S., Ragain J.C. (2005). Comparison of two video-imaging instruments for measuring volumetric shrinkage of dental resin components. J. Dent..

[30] Blackham J.T., Vandewalle K.S., Lien W. (2009). Properties of hybrid resin composite systems containing prepolymerized filler particles. Oper. Dent..

[31] Kleverlaan C.J., Feilzer A.J. (2005). Polymerization shrinkage and contraction stress of dental resin composites. Dent Mater..

[32] Lim B.S., Ferracane J.F., Sakaguchi R.L., Condon J.R. (2002). Reduction of polymerization contraction stress for dental composites by two-step light-activation. Dent Mater..

[33] Sarosi C., Moldovan M., Soanca A., Roman A., Gherman T., Trifoi A., Chisnoiu A.M., Cuc S., Filip M., Gheorghe G.F. (2021). Effects of monomer composition of urethane methacrylate-based resins on the C=C degree of conversion, residual monomer content and mechanical properties. Polymers.

[34] Petronijevic Sarcev B., Balos S., Markovic D., Sarcev I., Vukcevic M., Labus Zlatanovic D., Miletic V. (2021). Effect of the degree of conversion on mechanical properties and monomer elution from self-, dual- and light-cured core composites. Materials.

[35] Spahl W., Budzikiewicz H., Geurtsen W. (1994). Extractable residual monomers from various resin materials-a qualitative study. J Dent. Res..

[36] dos Santos R.L., de Sampaio G.A., de Carvalho F.G., Pithon M.M., Guênes G.M., Alves P.M. (2014). Influence of degree of conversion on the biocompatibility of different composites in vivo. J. Adhes. Dent..

[37] Ozturk B., Cobanoglu N., Cetin A.R., Gunduz B. (2013). Conversion degrees of resin composites using different light sources. Eur. J. Dent..

[38] Tapety C.M.C., Carneiro Y.K.P., Chagas Y.M., Souza L.C., Souza N.D.O., Valadas L.A.R. (2023). Degree of conversion and mechanical properties of a commercial composite with an advanced polymerization system. Acta Odontol. Latinoam..

[39] Atalı P.Y., Kaya B.D., Özen A.M., Tarçın B., Şenol A.A., Bayraktar E.T., Korkut B., Göçmen G.B., Tağtekin D., Türkmen C. (2022). Assessment of Micro-hardness, degree of conversion, and flexural strength for single-shade universal resin composites. Polymers.

[40] Ling L., Lai T., Malyala R. (2024). Mechanical properties and degree of conversion of a novel 3D printing model resin. Polymers.

[41] Tserki S.V., Papanastasiou G. (2002). Effect of chemical structure on degree of conversion in light-cured dimethacrylate-based dental resins. Biomaterials.

[42] Ling L., Xu X., Choi G.Y., Billodeaux D., Guo G., Diwan R.M. (2009). Novel F-releasing composite with improved mechanical properties. J. Dent. Res..

[43] Misev L., Schmid O., Udding-Louwrier S., Jong E.S.d., Bayards R. (1999). Weather stabilization and pigmentation of UV-curable powder coatings. J. Coatings Tech..

[44] Sideridou I.D., Karabela M.M., Vouvoudi E.C. (2008). Volumetric dimensional changes of dental light-cured dimethacrylate resins after sorption of water or ethanol. Dent. Mater..

[45] Örtengren U., Wellendorf H., Karlsson S., Ruyter I.E. (2001). Water sorption and solubility of dental composites and identification of monomers released in an aqueous environment. J. Oral Rehabil..

[46] Sideridou I., Tserki V., Papanastasiou G. (2003). Study of water sorption, solubility and modulus of elasticity of light-cured dimethacrylate-based dental resins. Biomaterials.

[47] Rahim T.N.A.T., Mohamad D., Akil H.M., Rahman I.A. (2012). Water sorption characteristics of restorative dental composites immersed in acidic drinks. Dent. Mater..

[48] Pereira S.G., Osorio R., Toledano M., Cabrerizo-Vílchez M.A., Nunes T.G., Kalachandra S. (2007). Novel light-cured resins and composites with improved physicochemical properties. Dent. Mater..

[49] Fonseca A.S.Q.S., Moreira A.D.L., de Albuquerque P.P.A.C., de Menezes L.R., Pfeifer C.S., Schneider L.F.J. (2017). Effect of monomer type on the CC degree of conversion, water sorption and solubility, and color stability of model dental composites. Dent. Mater..

[50] Ling L., Ma Y., Chen Y., Malyala R. (2022). Physical, mechanical and adhesive properties of novel self-adhesive resin cement. Int. J. Dent..

[51] Mizutani K., Takamizawa T., Ishii R., Shibasaki S., Kurokawa H., Suzuki M., Tsujimoto A., Miyazaki M. (2021). Flexural properties and polished surface characteristics of a structural colored resin composite. Oper. Dent..

[52] Barszczewska-Rybarek I.M. (2009). Structure-property relationships in dimethacrylate networks based on Bis-GMA, UDMA and TEGDMA. Dent. Mater..

[53] Alrahlah A., Khan R., Al-Odayni A.B., Saeed W.S., Bautista L.S., Alnofaiy I.A., De Vera M.A.T. (2023). Advancing dimethacrylate dental composites by synergy of pre-polymerized TEGDMA co-filler: A physio-mechanical evaluation. Biomimetics.

[54] Shibasaki S., Takamizawa T., Nojiri K., Imai A., Tsujimoto A., Endo H., Suzuki S., Suda S., Barkmeier W.W., Latta M.A. (2017). Miyazaki. M. Polymerization behavior and mechanical properties of high-viscosity bulk fill and low shrinkage resin composites. Oper. Dent..

